# Isolation and characterization of the human ovarian cell population for transplantation into an artificial ovary

**DOI:** 10.21451/1984-3143-AR2018-00140

**Published:** 2020-05-22

**Authors:** Parinaz Asiabi Kohneh Shahri, Maria Costanza Chiti, Christiani A. Amorim

**Affiliations:** Pôle de Recherche en Gynécologie, Institut de Recherche Expérimentale et Clinique, Université Catholique de Louvain, Brussels, Belgium.

**Keywords:** artificial ovary, cell isolation, endothelial cells, stromal cells

## Abstract

To support survival and growth of follicles, the transplantable artificial ovary should mimic the original organ, offering a physical (3D matrix) and biological support (cells). In order to replicate the ovarian cell populations, the aim of this study is to assess the proportions of stromal and endothelial cells in the ovarian cortex. To this end, ovarian biopsies were obtained from six women (mean age: 49 years). The epithelial layer and medulla were carefully removed. The cortex was finely minced and enzymatically digested and the isolated cells were fixed. For cell characterization, immunostaining for CD31 (for endothelial cells) and inhibin-α (for granulosa cells) was performed. Positive cells in each staining were counted and the proportion of the different cell populations was estimated from the total number of isolated cells. Since there is no specific marker for ovarian stromal cells, we estimated the proportion of these cells by performing a vimentin immunostaining and subtracting the proportions of CD31- and inhibin-α-positive cells. Immunostaining showed that 84% of isolated cells were vimentin-positive. From this pool, 3% were endothelial cells and 1% granulosa cells. Consequently, the population of ovarian stromal cells was 80%. In conclusion, our findings show that stromal cells represent the larger population of cells in the human ovarian cortex. While this ensures follicle survival and development in a normal ovary, we believe that the low proportion of endothelial cells could have a negative impact on the angiogenesis in the artificial ovary after the first days of transplantation.

## Introduction

The goal of a transplantable artificial ovary (TAO) is to mimic the natural ovary in order to support follicle survival and development and consequently restore fertility in cancer patients (Amorim and Shikanov, 2016). To do so, it should comply with two requirements: it must have a 3D physical structure for the isolated follicles (Amorim *et al*., 2009; Luyckx *et al*., 2013, 2014; Chiti *et al*., 2015) and some biological features, such as cell populations for angiogenesis, the synthesis of an extracellular matrix, and the formation of an ovary-like structure (Dath *et al*., 2011; Soares *et al*., 2015; Chiti *et al*., 2017b). 

In order to develop a suitable supporting matrix for human isolated follicles, different polymers have been used in our in vivo studies (Dolmans *et al*., 2007; Paulini *et al*., 2016; Chiti *et al*., 2018). Among all these studies, fibrin formulations with high concentrations of fibrinogen and thrombin were found to be the best choice for supporting follicle survival after xenotransplantation (Paulini *et al*., 2016; Chiti *et al*., 2017a, 2018). This is probably because their ultrastructure and rigidity resemble the human ovarian cortex (Paulini *et al*., 2016; Chiti *et al*., 2018).

Ovarian cells may also provide structural support in a TAO. Additionally, they are necessary because they show a complex bidirectional paracrine signaling with follicles (Tagler *et al*., 2011) and are responsible for vascularization in the ovarian tissue, which is also fundamental for follicle survival and development (Dath *et al*., 2011; Luyckx *et al*., 2014; Chiti *et al*., 2015). Dath *et al*. (2011) have demonstrated that when isolated human stromal and endothelial cells are grafted together, a well-organized and fully vascularized stromal structure was observed already after one week of xenografting in the murine ovarian bursa. Moreover, higher matrix degradation and formation of tissue that resembles the ovarian cortex were also observed. Contrarily, grafts without endothelial cells were smaller and poorly vascularized (Dath *et al*., 2011). Later, Soares *et al*. (2015), a study of xenografting of isolated human ovarian stromal and endothelial cells, led us to believe in a positive correlation between the number of grafted endothelial cells and vessel area after transplantation. Interestingly, Chiti *et al*. (2017b) also pointed out another positive correlation, this time between vessel area and follicle survival. If these hypotheses prove to be correct, we can presume that in order to improve follicle survival in a TAO, we should encapsulate a significant number of isolated endothelial cells. 

Our studies mentioned above have confirmed the significant role of grafting isolated ovarian cells, but, more important, they have also indicated that the number of endothelial cells seems to have an influence on the graft outcome. This led us to question our approach, described in Paulini *et al*. (2016), which was the grafting of 50,000 isolated human ovarian cells without taking into consideration the number of endothelial cells. Indeed, we do not know the proportions of stromal and endothelial cells in the human ovarian cortex soon after isolation. In order to answer this question, the aim of our study was to estimate the different cell populations obtained after the isolation procedure using biopsies of the human ovarian cortex.

## Methods

### Ethics

Use of human ovarian tissue was approved by the Institutional Review Board of the Université Catholique de Louvain on June 2, 2014 (IRB reference 2012/23MAR/125, registration number B403201213872). Use of human granulosa cells from aspirated antral follicles was approved by the Institutional Review Board of the Université Catholique de Louvain on January 12, 2015 (IRB reference 2014/16DEC/597, registration number B403201213872).

### Collection and dissection of ovarian tissue

After obtaining informed consent, fragments of ovarian tissue were collected from eight patients (mean age: 49 years) undergoing laparoscopic surgery for benign gynecological diseases. Ovarian biopsies were kept in DMEM + Glutamax^TM^-l (Dulbecco’s Modified Eagle Medium, Gibco, Paisley, UK) in an ice box while transferring from the hospital to the laboratory (less than 10 minutes). The medullary part of the tissue was removed with the help of scissors and the epithelial layer was carefully peeled with forceps. 

### Cell isolation procedure

The ovarian cortex was minced into small pieces with the help of a tissue chopper (McIlwain Tissue Chopper, Mickle Laboratory, Guildford, UK) adjusted to 0.5 mm. Next, tissue fragments were incubated in 10 ml Dulbecco's phosphate-buffered saline (D-PBS; BioWhittaker, Verviers, Belgium) containing 1 mg/ml collagenase type IA (Sigma, St Louis, USA). Collagenase digestion was performed in a water bath at 37°C for 45 minutes with gentle agitation and pipetted every 15 minutes. The enzymatic digestion step was halted by adding a similar volume of PBS, 10% heat-inactivated fetal bovine serum, and 1% Antibiotics-Antimycotic (Gibco). The cell suspension was then filtered through sterilized 80- and 11-μm nylon net filters (Millipore, Overijse, Belgium). The filtered solution was subsequently centrifuged at 180g for five minutes and the pellet resuspended in D-PBS and fixed for cytospin slides.

### Preparation of cytospin slides

Cytospin slides were prepared using Superfrost Plus slides (Menzel-Glaser, Braunschweig, Germany) and a Thermo Electron Cytospin 2 centrifuge (Fisher Scientific, Brussels, Belgium) operating at 700 rpm for five minutes. Each cytospin slide contained approximately 50,000 cells.

### Characterization of the ovarian cell population

Characterization of human isolated ovarian cells was assessed by immunohistochemical staining for 1) vimentin (mouse anti-human vimentin, clone V9, M072501-2, Dako, Heverlee, Belgium), 2) CD31 (mouse anti-human CD31, M0823, Dako) and 3) inhibin-α (MCA 951, Oxford Bio-Innovation, Langford Lane, UK) performed on cytospin slides. 

Vimentin is a cytoskeleton protein mainly expressed in the cytoplasm of cells of mesenchymal origin, and strongly expressed in ovarian stromal connective tissue (Czernobilsky *et al*., 1985). CD31/platelet endothelial cell adhesion molecule-1 (PECAM-1) immunostaining is mainly expressed on the human endothelial cells (Ensminger *et al*., 2002). Inhibin was assessed as a marker of granulosa cells (Dolmans *et al*., 2007). It has two forms, A and B, having the same α-sub unit but different β- subunits. The inhibin α-sub unit mainly expresses in all the granulosa cells of follicles at all stages of development (Bristol *et al*., 2004).

Cytospin slides with isolated ovarian cells were first rehydrated and permeabilized by immersing the slides in 0.05 M Tris-buffered saline (TBS 0.05M, pH 7.4) with 20% Triton X100 (Merck, Darmstadt, Germany). After blocking endogenous peroxidase activity with 0.3% H_2_O_2_ diluted in demineralized water, a demasking step was performed for 75 minutes at 98°C with 0.01 M citrate buffer. To block the non-specific antigen sites, slides were incubated with 0.05 M TBS, 10% normal goat serum and 1% bovine serum albumin for 30 minutes. Then the slides were incubated overnight at 4°C with primary antibodies: mouse anti-human vimentin (1:200), mouse anti-human CD31 (1:100) or mouse anti-human inhibin-α (1:100). Finally, slides were incubated with goat anti-mouse secondary antibodies conjugated to peroxidase (K4001, Dako) for one hour at 4°C. Diaminobenzidine was used as a chromogen (K3468, Dako). The slides were then counterstained with hematoxylin and mounted with DPX neutral mounting medium (Prosan, Merelbeke, Belgium). 

Negative control was stained with universal mouse negative control (N1698, Dako). Ovarian mesenchymal cells, isolated granulosa cells from human antral follicles, and human umbilical vein endothelial cells were used as positive controls for vimentin, inhibin-α and CD31, respectively. Pictures of four different areas in each slide were taken under the microscope (X200 magnification; Zeiss, Munich, Germany). In each area, 100 cells were counted and classified as positive (cells stained in brown) or negative (cells stained in blue). Proportions of endothelial and granulosa cells were calculated and used to estimate the population of ovarian stromal cells. 

### Statistical analysis

Comparisons of proportions of cells that were either positive or negative for vimentin, inhibin-α and CD31 were made using the chi-square test. Values of P < 0.05 were considered statistically significant.

## Results

### Characterization of the cell populations after isolation

**Figure 1:**
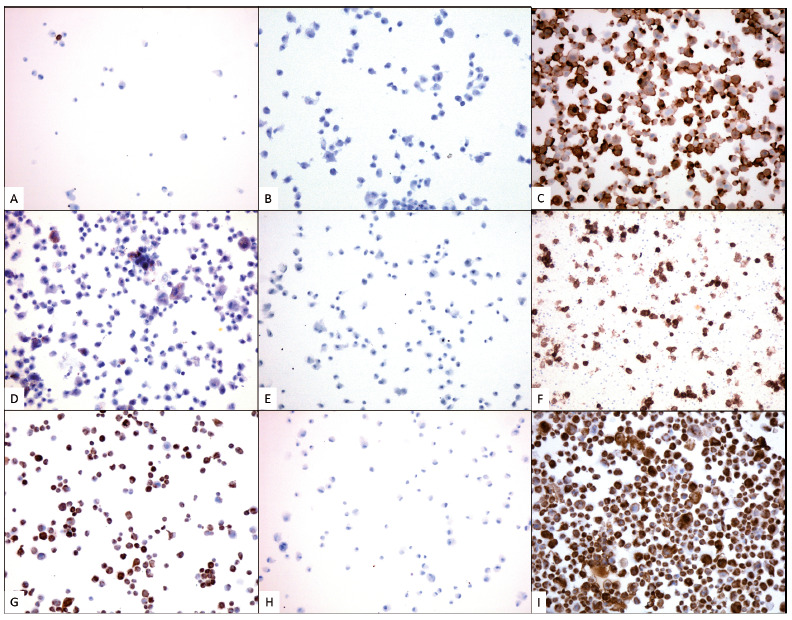
Immunostaining characterization of isolated human ovarian cells. Immunohistochemical staining (cells in brown) for CD31 (A), inhibin-α (D) and vimentin (G) and negative (B, E, H) controls. Commercially obtained HUVEC (C), granulosa (F) and ovarian mesenchymal cells (I) were used as positive controls for these three markers. All pictures are taken at X100 magnification.

Characterization of ovarian isolated cells was assessed with immunostaining with anti-human vimentin, CD31 and inhibin-α antibodies on cytospin slides (Fig. 1) prepared after cell isolation. Cell counting revealed 84% vimentin-positive cells (70-95% range), 3% CD31-positive cells (2-4%) and 1% inhibin-α-positive cells (0-2%). Table 1 shows the proportion of positive cells in each immunostaining in every patient. Considering that CD31- and inhibin-α-positive cells were also vimentin-positive, we can calculate the population of ovarian stromal cells by subtracting the proportion of these cells from the total proportion of vimentin-positive cells. Consequently, the proportion of ovarian stromal cells was estimated at 80%. Statistical analysis showed that the proportion of ovarian stromal cells was significantly higher than the proportions of granulosa and endothelial cells (P < 0.001). Moreover, granulosa cell population was statistically lower than that of endothelial cells (P < 0.001). 

**Table 1:** Proportion of vimentin-, CD31- and inhibin-α-positive cells in human ovarian cortex after cell isolation.

Patient	Age (years)	Vimentin-positive cells (%)	Inhibin-α-positive cells (%)	CD31-positive cells (%)
1	52	70	2	3
2	57	95	2	3
3	42	70	0	2
4	45	75	1	4
5	43	80	0	3
6	51	95	0	4

## Discussion

In TAO conception, in order to avoid inadvertent contamination of malignant cells, ovarian cells should be isolated from the remained ovary that went through chemo- or radiotherapy (Amorim, 2016). Since ovaries of patients after cancer treatment are atrophic and devoid of follicles, just like ovaries of menopausal women (Sklar *et al*., 2005; Overbeek *et al*., 2011), we decided to use ovaries of older patients in order to have an ovarian cortex similar to that of cancer survivors. While we predicted isolating a low number of granulosa cells due to the significant decrease in follicle population, we did not expect such a low proportion of isolated endothelial cells. 

To assess the role of endothelial cells in matrix vascularization, our previous studies identified this cell population using CD34 immunostaining (Dath *et al*., 2011; Soares *et al*., 2015). Our findings showed a proportion of approximately 16% of CD34-positive cells soon after isolation (Dath *et al*., 2011; Soares *et al*., 2015). While a higher proportion (18%) was reported in younger patients (34-49 years) (Soares *et al*., 2015), it does not seem statistically different from the percentage (14%) found in older women (42-78 years) (Dath *et al*., 2011). Independently of age, the percentage of CD34-positive cells reported in our previous studies was significantly higher than the proportion of CD31-positive cells found in this experiment. Since both CD31 and CD34 are markers for endothelial cells, we were surprised by such a discrepancy in our results. However, there are some possible explanations for the difference. For instance, it could be due to the fact that endothelial cells in different vessel types may express endothelial cell antigens heterogeneously (Pusztaszeri *et al*., 2006). Haber *et al*. (2015) reported that endothelial cells were only variably immunoreactive for CD31, while immunoreactivity for CD34 was more intense. Moreover, CD34 is expressed not only by endothelial cells, but also hematopoietic stem cells (Pusztaszeri *et al*., 2006). Unlike in our previous studies (Dath *et al*., 2011; Soares *et al*., 2015), in this present experiment we decided to assess CD31-positive cells because this transmembrane glycoprotein is a cell adhesion molecule with proangiogenic activity and has been shown to play an important role in angiogenesis (DeLisser *et al*., 1997; Zhou *et al*., 1999; Van Eyck *et al*., 2009), which is a necessary process after artificial ovary transplantation.

It is very likely that such a low proportion of CD31-positive cells after ovarian tissue dissociation is not enough to ensure rapid angiogenesis after grafting of the artificial ovary, which could negatively affect follicle survival and growth. In this case, an alternative could be increasing the number of CD31-positive cells by taking a biopsy that also contains the medullary part of the ovary. Indeed, Soares *et al*. (2015) showed that isolating endothelial cells from the ovarian medulla almost doubled the number of these cells compared to the ovarian cortex. In turn, this significantly increased the vessel area after transplantation of fibrin clots (Soares *et al*., 2015). It would be therefore important to devise a study to correlate the number of grafted isolated endothelial cells to artificial ovary vascularization and follicle survival after the first days of transplantation; for instance, a study where an artificial ovary prototype containing different proportions of endothelial cells associated with isolated human preantral follicles, stromal cells and lithium phthalocyanine (LiPc) crystals (Van Eyck *et al*., 2009; Manavella *et al*., 2018a) would be grafted into SCID mice. LiPc is very useful for in vitro and in vivo electron paramagnetic resonance oximetry because it is an oxygen-sensitive probe (Liu *et al*., 1993). Indeed, we already used LiPc to assess oxygenation in human ovarian tissue after xenotransplantation (Van Eyck *et al*., 2009; Manavella *et al*., 2018b). 

While isolating endothelial cells from the medulla of the remaining ovary could be a promising strategy for improving artificial ovary vascularization, for some cancer patients this may not be possible, as the ovary may become too atrophic after chemo-radiotherapy, or as both ovaries were removed. For them, an alternative could be the use of adipose tissue-derived stem cells. Manavella *et al*. (2018a,b) showed that adipose tissue-derived stem cells increases follicle survival by enhancing vascularization in xenografted frozen-thawed human ovarian tissue. They obtained a significantly higher pO_2_level in the adipose tissue-derived stem cells encapsulated in fibrin matrix with the ovarian tissue group compared to the ovarian tissue alone without adipose tissue-derived stem cells after seven days of xenografting. Interestingly, the total CD34-positive vessel area on day seven was greater in the human ovarian tissue xenografted with adipose tissue-derived stem cells. 

In conclusion, our findings confirm that the majority of cells found in the human ovarian cortex are stromal cells. While this ensures follicle survival and development in a normal ovary, we believe that in the artificial ovary, the low proportion of endothelial cells could have a negative impact on angiogenesis and consequently on follicle development after the first days of transplantation. In order to overcome this issue, we suggest isolating endothelial cells from the ovarian medulla (Soares *et al*., 2015) and including them in the TAO. This alternative could improve angiogenesis in TAO. However, our first step is to determine the right number of endothelial cells to be encapsulated in the artificial ovary.
